# Examining Predictors of Psychological Distress Among Youth Engaging with Jigsaw for a Brief Intervention

**DOI:** 10.1007/s10578-022-01436-2

**Published:** 2022-09-28

**Authors:** Niall Mac Dhonnagáin, Aileen O’Reilly, Mark Shevlin, Barbara Dooley

**Affiliations:** 1 School of Psychology, University College Dublin, Dublin, Ireland; 2 Jigsaw—The National Centre for Youth Mental Health, Dublin, Ireland; 3 School of Psychology, Ulster University, Coleraine, UK

**Keywords:** Youth, Mental health, Risk factors, Psychological distress

## Abstract

Risk factors for psychological distress among help-seeking youth are poorly understood. Addressing this gap is important for informing mental health service provision. This study aimed to identify risk factors among youth attending Jigsaw, a youth mental health service in Ireland. Routine data were collected from *N* = 9,673 youth who engaged with Jigsaw (Mean age = 16.9 years, SD = 3.14), including presenting issues, levels of psychological distress, age, and gender. Confirmatory Factor Analysis identified thirteen factors of clustering issues. Several factors, including *Self-criticism* and *Negative Thoughts,* were strongly associated with items clustering as psychological distress, however these factors were poorly predictive of distress as measured by the CORE (YP-CORE: R^2^ = 14.7%, CORE-10: R^2^ = 6.9%). The findings provide insight into associations between young people’s identified presenting issues and self-identified distress. Implications include applying appropriate therapeutic modalities to focus on risk factors and informing routine outcome measurement in integrated youth mental health services.

**Supplementary Information:**

The online version contains supplementary material available at 10.1007/s10578-022-01436-2.

## Introduction

Mental health is the leading health issue worldwide for youth between the ages of 12 and 25 years [1]. Common manifestations of youth mental health problems include anxiety, low mood or depression, self-harm, substance use, and behavioural problems [2, 3]. Youth mental health issues are associated with poorer educational and vocational outcomes [4], can negatively impact on personal relationships [5], and are a leading contributor to death by suicide [6]. Over time, youth mental health problems can develop into chronic mental health conditions in adulthood [7], with up to 80% of mental health conditions having an onset by age 26 [8]. Recent evidence from Canada, the United Kingdom, and Ireland indicates an increase in mental health problems among adolescents (defined in this study as ages 12 to 17) and young adults (ages 18 to 25), particularly among females [9–11]. Across these studies, the rise in mental health problems corresponds to increases in levels of anxiety, depression, self-harm, and suicidality. Together, these mental health problems are commonly known as manifestations of psychological distress [12, 13]. The rise in psychological distress among youth is a significant cause for concern in protecting their health, survival, and quality of life, which warrants an investigation into the risk factors for psychological distress in youth.

### Risk Factors

Previous research on risk factors has focused on personal psychological and behavioural risk factors, alongside contextual risk factors at different levels of young people’s ecological systems [14]. In previous research on depression and anxiety, the evidence base for psychological risk factors (e.g. self-criticism, perfectionism, repetitive negative thoughts, and rumination) [15–21] and behavioural risk factors (e.g. alcohol use and conduct problems) [10], has drawn typically from cross-sectional studies. Therefore, the evidence is insufficient to infer a causal relationship. However the evidence base is stronger regarding behavioural risk factors and self-harm, where longitudinal studies have shown that behavioural factors predict self-harm over time [22, 23]. Contextual risk factors identified previously include socio-demographic risk factors (e.g. low socio-economic status, minority sexual orientation (lesbian, gay, bisexual, asexual, pansexual; LGBAP), ethnic minority status, female gender) [24–27], family risk factors (e.g. adverse childhood experiences, high levels of parental criticism) [10, 28], interpersonal risk factors (e.g. bullying, issues with peers and romantic relationships) [29–31], poor connectedness in school and long-term unemployment [32–34]. However, research has typically focused on isolated risk factors, with few studies including multiple risk factors to predict psychological distress in youth.

A subsample of the general population of youth who are at high risk of psychological distress are help-seeking youth attending mental health services in primary care. For example, evidence from headspace Australia—a youth mental health service established in 2006 [35] —indicates that 69% of attendees to the service display high levels of psychological distress, compared to 9% of youth in the general population [36]. Yet, what is known about risk factors for psychological distress among youth largely draws from studies conducted in the general population [10, 24, 27, 37] rather than through research with help-seeking youth who demonstrate a higher level of risk. Although some studies have focused on risk factors for self-harm or on prior suicidality as a predictor of outcomes in help-seeking youth [38, 39], these studies did not provide insight into psychological, behavioural, and contextual factors predicting anxiety and depression. An examination of the patterns between risk factors and psychological distress specifically for help-seeking youth could inform service provision, helping to meet the needs of youth and improve mental health outcomes.

### Integrated Youth Mental Health Services

The past 15 years has seen the development of integrated youth mental health services (IYMHS) internationally. These are services that are youth-friendly, evidence-informed and accessible for young people [40, 41]. Typically, these services provide mental health support at primary care [42]. Examples include headspace Australia, Israel [35, 43], ACCESS Open Minds (Canada) [44], and Jigsaw (Ireland) [45]. Research to date has indicated that anxiety and depression or low mood are typically the most common presenting issues among help-seeking youth [36, 46–49]. However, young people are likely to attend services with more than one presenting issue, with previous research identifying self-criticism, anger, substance use, and a range of contextual issues as common presenting issues [47, 50–52]. In addition, the co-occurrence of youth mental health difficulties is well-established in previous research (e.g. co-occurrence of suicidal ideation and behavioural difficulties among youth) [53].Yet few studies have examined relationships between presenting issues [48], either to identify how presenting issues co-occur, or to identify whether there are clear factors underlying presenting issues that could be understood as risk factors for psychological distress among help-seeking youth.

### The Present Study

The present study examines psychological, behavioural, and contextual risk factors for psychological distress among youth engaging with Jigsaw for brief intervention support, by focusing on the issues young people present with when first engaging with the service. The first aim of the study is to examine the associations between presenting issues among youth engaging with Jigsaw to identify factors theoretically mapped to psychological, behavioural, or contextual domains of youth mental health. The second aim is to examine associations between identified factors and manifestations of psychological distress (e.g. anxiety, depression, self-harm). The third aim is test whether the factors predict psychological distress among help-seeking youth.

## Method

### Participants and Procedure

The sample consisted of *N* = 9673 young people who had engaged with Jigsaw for a brief intervention between September 2013 and February 2019. Brief intervention support consisted of up to eight therapeutic sessions one-to-one with a Jigsaw clinician. The first session was an initial screening session, involving a conversation between the young person and clinician to determine whether the brief intervention was appropriate for the young person’s needs. The second session was an assessment session, following a structured interview on the young person’s home, education, activities, drug and alcohol use, sexuality, and signs of mental health difficulties (HEADSS) [54]. The remaining six sessions were therapeutic intervention sessions.

Inclusion criteria were young people aged 12–25 who had engaged with brief intervention support with Jigsaw and had completed an age-appropriate measure of psychological distress. Young people outside of this age range who had not completed a measure were excluded from the sample. Information about youth engaging with Jigsaw was captured by staff using the Jigsaw data system (JDS), an electronic case management and evaluation tool. Young people (and parents/guardians, where required) in this study provided informed consent to their data being collected and stored for research purposes. The study received ethical approval from the first author’s host institution (reference no. HS-E-19-30).

### Measures

Information gathered included age, gender, presenting issues and levels of psychological distress. Psychological distress scores were gathered by clinicians in the initial screening session, whereas presenting issues were typically inputted onto the JDS by clinicians following the initial screening and the assessment session. It was possible for clinicians to add further presenting issues to the JDS throughout the intervention. Presenting issues consisted of 123 binary items listed on the JDS, nested under eight categories (*Thinking, Interaction, Physical, Feelings, Behaviour, School/Work, Family/Peers/Relationships, Community/Setting*), with an open-ended space available under each category to describe additional presenting issues (a list of all presenting issues with information on prevalence is included in Table S1, Supplementary Materials). Therefore presenting issues included but were not limited to a young person’s mental health difficulties, with some presenting issues related to the young person’s broader social context. Presenting issues were gathered based on the information the young person had shared with the clinician in the initial screening and assessment, but there were no standard definitions of the presenting issues provided to clinicians. Presenting issues were gathered on the JDS to assist clinicians with case management and for the purposes of research and evaluation in Jigsaw.

Presenting issues identified by clinicians were coded “Yes” on the JDS and items that were not selected were coded “No” by default. As such, there were no missing data among the presenting issues. The content of open-ended presenting issues was examined and, where appropriate, re-coded into existing binary variables. In addition, where issues were observed to occur with some frequency, new presenting issue items were generated (e.g. *Physical Discomfort and Sickness*). Presenting issues with very low prevalence (i.e. < 1%) were omitted, yielding a final set of 82 presenting issue items for the analysis.

Psychological distress was measured via the Clinical Outcome in Routine Evaluation (CORE) measures; either the YP-CORE [55] (for age 12 to 16 years; *N* = 5007) or the CORE-10 [56] (for ages 17 to 25 years, *N* = 4666). Both are ten-item measures that have demonstrated high levels of inter-item reliability and unidimensional factor structures in samples of help-seeking youth [57]. Both scales showed strong reliability in this study (α_YP-CORE_ = 0.812, α_CORE-10_ = 0.774). Regarding missing data, only young people who completed all ten items of a CORE measure (*N* = 9664) or had only one item missing (*N* = 9) were included in the sample. Guidelines for the CORE measures were followed whereby a total mean score was calculated for young people who completed nine items [58].

### Data Analysis

To address the first aim of the study, exploratory factor analysis (EFA) and confirmatory factor analysis (CFA) were conducted on the data to identify factors underlying youth presentations to Jigsaw. The dataset was randomly split in half to perform EFA and CFA. EFA models with 1 through to 14 factors were estimated using Weighted Least Squares Means- and Variances-adjusted (WLSMV) to allow for the analysis of binary data, under the assumption of bivariate normality underlying the presenting issues [59], and Oblique (Geomin) rotation was applied, allowing factors to correlate. Fit indices were used to assess the relative and absolute fit of all the models, summarised in Table 1, where thresholds were in line with previous research [59–62]. Models with acceptable model fit were then tested using CFA.

**Table 1:** Fit indices for factor analyses

Fit index	Acceptable fit	Excellent fit
Relative-χ^2^ (chi-square/degrees of freedom)	≤ 3	≤ 2
Root mean square error of approximation (RMSEA)	≤ 0.08	≤ 0.06
Comparative fit index (CFI)	≥ 0.90	≥ 0.95
Tucker lewis index (TLI)	≥ 0.90	≥ 0.95
Standardised root mean square residual (SRMR)	≤ 0.08	N/A

The Root Mean Squared Error of Approximation (RMSEA) and Standardised Root Mean Square Residual (SRMR) were examined with caution, as both indices are affected by sample size [63]. For factor loadings (λ), λ ≥ 0.3 was considered acceptable [64], with items with λ < 0.3 removed. At the factor level, Average Variance Explained (AVE) was calculated to compare convergent validity across factors, and Joreskog’s Rho (ρ_η_) ≥ 0.7 was set as the threshold for composite reliability [65]. Factor analyses were conducted on Mplus 8.4 [66].

To address the second aim, Pearson correlations were conducted using factor scores to examine associations between individual factors derived from the factor analysis and psychological distress. For the third aim, multiple regression analyses were conducted to examine the predictive power of multiple factors on psychological distress. To assess multicollinearity, tolerance values of ≥ 0.1 were considered acceptable for the multiple regression analysis [64]. Pearson correlations and multiple regression analyses were performed on SPSS 24 [67].

## Results

### Description of the Sample

Demographic information captured included age, gender, family of origin, living circumstance, and education and employment status. However, due to high levels of missing data on most demographic factors the analysis was limited to age and gender, whereby 60.6% of participants were female and 39.4% were male. The mean age of participants was 16.9 years (SD = 3.14). Of the 82 presenting issue items, the most common presenting issues in the final sample were *Anxiety* (53%), *Low Mood* (39.7%), and *Stress* (29.8%).

### EFA

EFA was conducted on the 82 items, generating 14 models. Models with seven factors or more met the criteria for excellent fit on most indices (χ^2^/df range = (1.059, 1.357), RMSEA = (0.003, 0.009), comparative fit index (CFI) = (0.952, 0.993), Tucker Lewis index (TLI) = (0.942, 0.99), SRMR = (0.059, 0.078)). The factors generated included descriptions of psychological distress (*Feeling Anxious, Feeling Depressed/Withdrawing, Self-harm and Suicidality*), psychological factors (*Self-criticism, Negative Thoughts*), behavioural factors (*Acting Out, Risky Behaviour*), and contextual factors (*Problems with Family, Problems with Peers, Physical Health and Eating, School Problems, Trauma, Lack of Support Structures*). Factors *Self-harm and Suicidality, Feeling Anxious, Problems with Peers, Acting Out, Problems with Family, Risky Behaviour,* and *Feeling Depressed/Withdrawing* were generated in all models. The 14-factor model included one factor which did not align with theory and this factor was not included in any further analysis.

Based on the findings of the EFA, six models were identified for the next stage of CFA, ranging from an eight-factor model to a 13-factor model. As items were removed following EFA due to low factor loadings, the number of items included ranged from 52 to 71 items out of 82 presenting issue items. The composition of factors is summarised in Table 2.

**Table 2:** Composition of factors with factor loadings from CFA with variance explained and reliability estimates

Factor	AVE	Composite reliability (ρ_η_)	Item	Factor loading
Feeling anxious	58.23%	0.892	Anxiety	0.894
			Heart racing	0.828
			Chest pain or heaviness	0.771
			Panic attacks	0.720
			Dizzy/lightheaded	0.703
			Nausea	0.634
Self-harm and suicidality	53.94%	0.823	Suicidal thoughts/gestures	0.803
			Thoughts of hurting self	0.796
			Suicide attempt	0.671
			Self-injurious behaviour	0.655
Risky behaviour	46.85%	0.81	Use of alcohol	0.807
			Risk-taking	0.788
			Use of drugs	0.696
			Negative peer involvement	0.602
			Impulsiveness	0.473
Negative thoughts	46.61%	0.771	Unpleasant thoughts	0.773
			Recurring thoughts	0.748
			Thoughts racing	0.708
			Obsessions/compulsions	0.454
Acting out	42.77%	0.862	Aggressiveness	0.829
			Anger	0.795
			Arguing	0.769
			Easily irritated	0.744
			Frustration	0.650
			Behaviour problems	0.629
			Mood swings	0.572
			Impulsiveness	0.388
			Parent/guardian/youth conflict*	0.299
Self-criticism	45.52%	0.799	Self-criticism	0.796
			Feeling worthless	0.789
			Low self-esteem	0.704
			Feeling guilty or shameful	0.608
			Perfectionistic	0.393
Physical health and eating	38.54%	0.783	Eating difficulties	0.784
			Weight problems	0.728
			Pain	0.683
			Physical discomfort, sickness	0.520
			Health problems	0.474
			Appetite changes/issues*	0.454
Feeling depressed/withdrawing	34.02%	0.81	Low energy	0.737
			Low mood	0.721
			Feeling hopeless	0.715
			Lack of interest	0.676
			Sleep changes/issues	0.618
			Excessive crying	0.519
			Isolating from others/withdrawal*	0.420
			Appetite changes/issues*	0.358
			Poor concentration*	0.274
Lack of support structures	32.45%	0.691	Lack of family social support*	0.659
			Lack of supportive adults*	0.642
			Lack of social support*	0.614
			Unemployed young adult	0.578
			Family financial problems*	0.255
Trauma	29.07%	0.58	Experienced trauma	0.729
			Bad dreams/nightmares	0.681
			Grief/bereavement	0.333
			Acute family crisis*	0.238
Problems with peers	28.96%	0.752	Loneliness	0.759
			Trouble making/keeping friends	0.674
			Bullied	0.558
			Shyness	0.546
			Feeling ignored or abandoned*	0.525
			Relationship-peers	0.429
			Lack of social support*	0.343
			Isolating from others/withdrawal*	0.316
Problems with family	27.35%	0.788	Family problems	0.824
			Family violence	0.718
			Parent/guardian substance abuse	0.646
			Parent/guardian mental illness	0.577
			Family financial problems*	0.466
			Marital/relationship problems	0.440
			Parent/guardian/youth conflict*	0.428
			Feeling ignored or abandoned*	0.390
			Acute family crisis*	0.368
			Lack of supportive adults*	0.345
			Lack of family social support*	0.252
School problems	24.82%	0.654	Procrastination	0.608
			Confusion	0.551
			Academic problems	0.541
			Poor concentration*	0.528
			Learning disability	0.402
			Transition problems	0.288

*AVE* average variance explained

*Items cross-loading onto more than one factor

### CFA

On the model fit statistics, the 13-factor model demonstrated superior fit to other models in the CFA. This model was the only model to meet criteria for excellent fit on the χ^2^/df (1.944), and demonstrated the highest CFI (0.902). This model did not meet the criteria for good fit on the TLI (0.895) or SRMR (0.096), but the TLI was higher than that of other models and marginally below the threshold of acceptable fit. Due to the superior performance of this model, all further analyses were conducted on this model only.

Most factor loadings were above the threshold for good fit (λ > 0.3; see Table 2). In general, loadings below the threshold for this model were for items cross-loading onto more than one factor, with the exception of *Transition Problems* (*School Problems*). Looking to fit statistics at the factor level, 10 out of 13 factors demonstrated good composite reliability, with *Feeling Anxious* demonstrating the strongest reliability and AVE (see Table 2).

### Associations Between Factors and Psychological Distress

Pearson correlations between factor scores indicated the likelihood of particular types of presentations to co-occur with presentations of psychological distress. In general, correlations with *Feeling Anxious* were weaker than with *Feeling Depressed/Withdrawing* and *Self-harm and Suicidality*. Psychological factors (*Self-criticism, Negative Thoughts*) were most strongly associated with *Feeling Depressed/Withdrawing* (*r* = 0.807, 0.712 respectively) and *Feeling Anxious (r* = 0.492, 0.585 respectively). However, correlations between behavioural factors and *Feeling Anxious* were either very weak (*Risky Behaviour, r* = 0.025) or had a negatively-directed relationship that was weak (*Acting Out, r* = − 0.105). The factors most strongly associated with *Self-harm and Suicidality* were *Feeling Depressed/Withdrawing* (*r* = 0.675)*, Self-criticism* (*r* = 0.610), and *Risky Behaviour* (*r* = 0.599).

Contextual factors were most strongly associated with *Feeling Depressed/Withdrawing*. All contextual factors apart from *Problems with Family* were strongly correlated with *Feeling Depressed/Withdrawing* (all *r* > 0.6), where the strongest association was with *School Problems* (*r* = 0.717). *Trauma*, *Physical Health and Eating,* and *Problems with Peers* were the contextual factors most closely associated with *Self-harm and Suicidality* (*r* = (0.536, 0.550))*.* Associations with *Feeling Anxious* were weaker among the contextual factors*.* The contextual factor most closely associated with *Feeling Anxious* was *Physical Health and Eating* (*r* = 0.481). Correlations between psychological distress factors and all other factors are presented in Table 3, and a correlation matrix for all factors is included in Table S2 in supplementary materials.

**Table 3:** Correlations between psychological distress factors and other factors

	Feeling anxious	Feeling depressed/withdrawing	Self-harm and suicidal behaviours
Feeling anxious	1		
Feeling depressed/withdrawing	0.417***	1	
Self-harm & suicidality	0.151***	0.675***	1
Negative thoughts	0.585***	0.712***	0.510***
Self-criticism	0.492***	0.807***	0.610***
Acting out	− 0.105^***^	0.322***	0.386***
Risky behaviour	0.025*	0.445***	0.599***
Problems with family	0.030**	0.424***	0.410***
Problems with peers	0.301***	0.637***	0.550***
Physical health and eating	0.481***	0.692***	0.536***
School problems	0.274***	0.717***	0.432***
Trauma	0.252***	0.654***	0.546***
Lack of support structures	0.179***	0.688***	0.495***

***p < 0.001, **p < .01, *p < .05

### Multiple Regression Analysis

When addressing the third aim in the analyses, age and gender were included in the regression model. This was to examine whether the predictive power of factors to levels of psychological distress could be explained over and above the contribution of age and gender to levels of psychological distress. As the outcomes of interest in the regression were levels of psychological distress as per the CORE measures, the psychological distress factors identified earlier in the analyses (*Feeling Anxious, Feeling Depressed/Withdrawing, Self-harm & Suicidality*) were omitted from the regression analysis to avoid conceptual overlap.

Age, gender, and the factors were poor predictors of levels of psychological distress on both the YP-CORE and CORE-10. The model predicted 14.7% of variance in YP-CORE scores (*F*(12, 4994) = 71.796, *p* < 0.001) and 6.9% of variance in CORE-10 scores (*F*(12, 4653) = 28.678, *p* < 0.001). All tolerance estimates were acceptable (> 0.1) indicating low levels of multicollinearity. The significant predictors of YP-CORE scores were female gender, older age, *Self-criticism*, and *Physical Health and Eating*. The most significant predictors of CORE-10 scores were female gender, *Physical Health and Eating*, older age, and *Risky Behaviour*. Although estimates were statistically significant, the contributions of each factor to YP-CORE or CORE-10 scores were low (β = (− 0.142, 0.204)) (Table 4).

**Table 4:** Multiple regression

Factor	YP-CORE			
	*B*	SE	β	*t*
(Constant)	1.933	1.145		1.687
Gender (female)	3.097	0.206	0.204	15.038***
Age	0.699	0.076	0.122	9.217***
Negative thoughts	− 0.785	0.628	− 0.047	− 1.251
Self-criticism	4.635	0.942	0.174	4.922***
Acting out	− 0.433	0.368	− 0.033	− 1.177
Risky behaviour	− 0.177	0.510	− 0.008	− 0.348
Problems with family	− 1.645	0.977	− 0.057	− 1.684
Problems with peers	0.156	0.602	0.007	0.259
Physical health and eating	2.256	0.499	0.079	4.517***
School problems	1.323	0.622	0.051	2.128*
Trauma	1.568	0.791	0.071	1.983*
Lack of support structures	0.709	0.829	0.029	0.856

Factor	CORE-10			
	*B*	SE	β	*t*
(Constant)	11.350	0.862		13.161***
Gender (female)	1.356	0.195	0.101	6.968***
Age	0.220	0.041	0.078	5.371***
Negative thoughts	− 1.489	0.535	− 0.114	− 2.786**
Self-criticism	1.887	0.818	0.089	2.308*
Acting out	− 1.357	0.354	− 0.100	− 3.835***
Risky behaviour	1.568	0.367	0.106	4.270***
Problems with family	0.394	0.842	0.016	0.468
Problems with peers	1.915	0.613	0.097	3.125**
Physical health and eating	2.922	0.474	0.121	6.159***
School problems	1.082	0.550	0.050	1.968*
Trauma	1.900	0.642	0.116	2.962**
Lack of support structures	− 2.448	0.626	− 0.142	− 3.910***

***p < 0.001, **p < .01, *p < .05

## Discussion

This study identified 13 factors underlying presentations to Jigsaw, including psychological factors (*Self-criticism, Negative Thoughts*), behavioural factors (*Acting Out, Risky Behaviour*)*,* contextual factors (*Problems with Family, Problems with Peers, Physical Health and Eating, School Problems, Trauma, Lack of Support Structures*)*,* and manifestations of psychological distress (*Feeling Anxious, Feeling Depressed/Withdrawing, Self-harm and Suicidality*)*.* Although the factors were poor predictors of the intensity of psychological distress as measured by the CORE and YP-CORE, psychological, behavioural, and contextual factors were associated with presentations of *Feeling Anxious, Feeling Depressed/Withdrawing,* and *Self-harm and Suicidality*. This may have implications for service provision with youth, as this study highlights areas where youth can be supported with their mental health. In addition, the findings can inform data capture practices in IYMHS, including identifying presenting issues that accurately reflect the issues facing young people and informing routine outcome measurement in services.

Presentations of *Feeling Anxious, Feeling Depressed/Withdrawing,* and *Self-harm and Suicidality* were all associated with psychological factors *Self-criticism* and *Negative Thoughts.* This supports previous cross-sectional studies where self-criticism and repetitive negative thinking were associated with psychological distress in youth [16–18], and shows that this association is strong among help-seeking youth. In previous research with adults, perfectionism and repetitive negative thinking have been identified as transdiagnostic risk factors, rather than factors that predict one specific type of mental health problem [68, 69]. As *Self-criticism* and *Negative Thoughts* were associated with each of *Feeling Anxious, Feeling Depressed/Withdrawing* and *Self-harm and Suicidality* in this study, this may reflect on the transdiagnostic nature of these risk factors for youth, although further research with youth is warranted.

In identifying an association between *Self-criticism, Negative Thoughts,* and manifestations of psychological distress, there are potential implications for service provision with youth. To help reduce negative thinking and self-criticism, therapeutic modalities such as Cognitive Behavioural Therapy (CBT) [70, 71] and Compassion-focused Therapy (CFT) [72] could be applied in brief interventions. IYMHS have commonly endorsed the use of CBT in brief interventions [42], but CFT is less commonly noted in IYMHS research to date [45].

Behaviourally, *Risky Behaviour* and *Acting Out* were most closely associated with *Self-harm and Suicidality*, whilst also showing strong associations with *Feeling Depressed/Withdrawing*. The relationship between behavioural factors and self-harm and suicidal behaviours is well-established in previous research [22, 23, 53] and supported by the findings of this study. Overall, this suggests that clinical staff should be mindful of behavioural presenting issues such as anger, alcohol use, and substance use among help-seeking youth as potential indicators of risk of *Self-harm and Suicidality*, or of *Feeling Depressed/Withdrawing*. Indeed, behavioural risk factors are an under-researched area in IYMHS, where routinely-used outcome measures such as the CORE measures [55, 56] or the Kessler Distress Scale [73] typically do not pick up on behavioural issues affecting youth. Ongoing research in Orygen and headspace is underway to identify a wide set of standardised measures relevant to youth presentations [46]. The presence of behavioural presenting issues in this sample highlights the potential need to measure issues such as anger, and drug and alcohol misuse in help-seeking youth alongside measuring psychological distress.

Most contextual factors were strongly associated with *Feeling Depressed/Withdrawing*. *Problems with Peers* was most strongly associated with *Feeling Depressed/Withdrawing* and *Self-harm and Suicidality*, supporting previous research in the general population which identified bullying, isolation, and difficult peer relationships as risk factors for psychological distress [29, 30]. For *School Problems*, youth presenting with procrastination, confusion, and general academic problems were also likely to present with signs of *Feeling Depressed/Withdrawing.* Together, these findings point to the school environment as an important area for supporting youth through anti-bullying strategies, supporting isolated youth, and young people experiencing difficulties with schoolwork, all of whom are vulnerable to developing psychological distress. The identification of a *Trauma* factor, again associated most strongly with *Feeling Depressed/Withdrawing* and *Self-harm and Suicidality* supports previous research on adverse childhood experiences (ACEs) as predictors of psychological distress [28]. In the context of providing brief intervention support, this reinforces the need for IYMHS to consider trauma-informed approaches when working with youth, which has been identified in national mental health policy in Ireland [74]. *Physical Health and Eating* was among the strongest predictors of *Feeling Anxious, Feeling Depressed/Withdrawing,* and *Self-harm and Suicidality*. The strong associations identified in this study may be explained in part by the co-morbidity between eating disorders, anxiety, low mood, and self-harm established in previous youth research [75].

Notably, the associations between psychological, behavioural, and contextual factors and presentations of psychological distress did not translate into predicting the intensity of psychological distress in this study. The nature of data capture in the study may have contributed in part to this issue. Young people’s presenting issues were captured as binary variables, and the factors were generated based on these data. However, many of these presenting issues have been studied as continuous variables in previous youth research [10, 17]. The reduction of these issues into binary data may have affected the predictive validity of the data. For example, young people who presented with perfectionism may have had mild, moderate, or severe presentations relating to this issue, and a binary variable did not reflect the dimensional nature of the underlying construct for this and similar presenting issues. Overall, this may have had implications for the predictive power of the factors on psychological distress.

### Strengths, Limitations, and Future Directions

Strengths of this study include the large sample of help-seeking young people and the breadth of presenting issues gathered. Limitations include statistical restrictions in working with binary data where presenting issues had low prevalence, issues of clarity with some presenting issues, high levels of missing data among demographic variables, the cross-sectional nature of the data, and the focus on risk factors only without the consideration of protective factors for youth mental health. The benefits and shortcomings relating to each of these issues are discussed below with suggestions for future research and data capture practices with IYMHS.

One of the key strengths of this study is in the use of a large dataset which was routinely collected in an IYMHS. This allowed for an analysis of patterns of presenting issues and psychological distress in over 9000 help-seeking youth which builds on previous research with Jigsaw [47, 48], and adds to international research in improving our understanding of youth engagement with IYMHS [36, 50, 51]. Mental health research is a field that has typically been plagued with issues of small samples leading to analyses that are underpowered [76]. The use of routine outcome measurement in mental health services provides an opportunity to address this issue, by gathering data naturalistically with help-seeking individuals as they engage with services [77].

A further strength of this dataset was in the breadth of presenting issues recorded. This allowed for the identification of factors that reflected on issues underlying presentations to IYMHS. Yet the heterogeneity of the presenting issues posed a challenge for measuring the predictive power of risk factors on psychological distress, as many presenting issues had low levels of prevalence. Of the original 123 presenting issues in the dataset, 82 issues were prevalent in more than 1% of the sample which were included in this analysis, and only 21 issues were prevalent in more than 10% of the sample. This led to statistical restrictions in this study, as low prevalence can reduce the accuracy of a variable predicting a given outcome [39]. In future, IYMHS might consider reducing the number of presenting issues on their data systems, which could be informed by the factors generated in this study (e.g. *Panic Attacks, Heart Racing,* and *Anxiety* could all merge under one *Feeling Anxious* factor), leading to a smaller number of concise presenting issues that more accurately predict psychological distress.

Yet there is also a case for the inclusion of other presenting issues or renaming presenting issues to clarify the nature of the issues affecting help-seeking youth. LGBAP sexuality, for example, has been identified in previous research as a risk factor for psychological distress [25]. In the dataset used in this study, sexual orientation was not captured, and the presenting issue labelled *Sexual Identity Issues* had very low prevalence. The label for this presenting issue was not sufficient to capture the diversity of sexual orientations among young people, which may have contributed to the low prevalence. Measurement of gender in this dataset was limited to male and female, although subsequent updates to the JDS are more inclusive as they allow young people to specify their gender identity. In addition, demographic variables showed high levels of missingness, possibly because it was not compulsory for staff to complete these sections on the JDS. This meant important equity-related variables, such as living circumstance, family of origin, and education and employment status could not be included in the analysis. To improve on this in future research, consistency may be achieved by allowing young people to complete the demographic questions themselves, for example while they are in a waiting room or at the beginning of a session with the assistance of their clinician.

Due to the cross-sectional nature of the data, the relationships identified between risk factors and psychological distress do not provide insight into causality, instead highlighting co-existence between particular presentations and manifestations of psychological distress. Future research could potentially focus on longitudinal outcomes based on presentations to IYMHS, although the findings from this study demonstrate the methodological difficulties in establishing those associations.

Finally, the focus of the present study was on risk factors only, but young people also experience protective factors in areas such as family, peers, school, along with having personal strengths that were not measured in this study [14]. In future, data systems could also capture young people’s strengths and protective factors, helping to provide a broader picture of the factors that are buffers against psychological distress alongside capturing risk factors.

## Summary

This study identified 13 factors underlying young people’s presentations to Jigsaw, an IYMHS in Ireland, by analysing a large dataset of young people who had engaged with Jigsaw for a brief intervention. Factors were identified that were strongly associated with presentations of psychological distress, including *Self-criticism, Negative Thoughts, Physical Health and Eating* and *Risky Behaviour.* By identifying risk factors among a large sample of help-seeking youth, the findings provide insight into areas in which clinical staff may focus on during an intervention to help young people who are in distress. Furthermore, the challenges and limitations identified in this study in gathering young people’s presenting issues and demographic information can inform IYMHS in improving routine outcome measurement and data capture with youth. Overall, this study provides a novel contribution to the growing field of IYMHS, which prioritises the application of evidence-informed mental health support to young people.

### Supplementary Information

Below is the link to the electronic supplementary material.

Supplementary file1 (XLSX 17 kb)

## Data Availability

Participants of this study did not agree for their data to be shared publicly.

## Code Availability

Code and software outputs from this study are available on the Open Science Framework: https://osf.io/g2znu/?view_only=e9f7d864c7dc40f4b9eca610c436f931
